# Characterizing the dielectric properties of carbon fiber at different processing stages

**DOI:** 10.1038/s41598-021-96949-6

**Published:** 2021-09-01

**Authors:** Hsien-Wen Chao, Hung-Chun Hsu, Yen-Ren Chen, Tsun-Hsu Chang

**Affiliations:** 1 Department of Physics, National Tsing Hua University, Hsinchu, 300 Taiwan; 2 Department of Material Science and Engineering, National Tsing Hua University, Hsinchu, 300 Taiwan

**Keywords:** Polymers, Electronic properties and materials, Characterization and analytical techniques

## Abstract

The polyacrylonitrile (PAN) fibers go through a series of chemical reactions in various processing temperatures/stages and finally turn into the so-called carbon fibers. Oxidization is the first stage, and it takes the largest proportion of the entire processing time for the tremendous change from a chain texture to a ladder texture. The pre-carbonization of carbon fibers is then achieved using a furnace with a higher processing temperature (typically at 700–900 °C). During the reaction processes, the color of the fibers changes from white (PAN) to light black (oxidation), and eventually to black (pre-carbonization). Characterizing the complex permittivity helps us determine the carbonization status of the fibers. This work employed the enhanced-field method (EFM) and the contour mapping method to determine the fibers’ dielectric properties for the first time. Results show that both the real and imaginary parts of permittivity increase as the processing temperature rises. The dielectric constants change from 2.82 (PAN) to 6.50 (pre-carbonization), and the loss tangents increase from 0.007 (lossless) to 0.089 (lossy). This study provides a simple and effective method for characterizing carbon fibers’ processing status and can be applied to the measurement of other fibrous materials.

## Introduction

Carbon fiber, an advanced composite material, features lightweight, low thermal expansion, high stiffness, high tensile strength, high-temperature tolerance, and high chemical resistance. Carbon fibers have numerous applications, such as aerospace, automotive, wind turbine, and sports equipment^1,2^. Polyacrylonitrile (PAN) is a commonly used raw material, also known as a precursor, to fabricate high-quality carbon fibers with high tensile strength and modulus. The precursor, i.e., PAN, is heated to approximately 200–300 °C in the air to break the hydrogen bonds and oxidize the original chain texture, called the oxidization. The oxidized fibers are then heated to a higher temperature, which drives off the non-carbon atoms like oxygen and nitrogen during the pre-carbonization and post-carbonization stages. The carbonized fiber processes under an even higher temperature and produces the ultra-high modulus carbon fiber (graphitization)^3,4^.

Many measurements are introduced to distinguish the properties of carbon fiber in different stages, such as FTIR (Fourier Transform Infrared Spectroscopy), DSC (Differential Scanning Calorimeter), XRD (X-ray Diffraction), and SEM (Scanning Electron Microscope)^5–9^. FTIR records the infrared absorbance spectrum of samples and distinguishes the variety of chemical band structures. DSC measures the difference in the amount of heating flow required between samples and reference, distinguishing the oxidation stage^5^. XRD detects the proportion of crystalline structures of the samples and distinguishes the chemical structure transformation progress^9,10^. SEM scans the surface of samples, showing the sheath-core structure, which visualizes the structure transformation^5,10^. Besides, FTIR, DSC, and XRD measurements can also be used to estimate the aromaticity index (AI%) for determining the degree of oxidization^11^. The existing measuring techniques are expensive and time-consuming, and have to determine the fiber’s properties jointly. Here we proposed a new and efficient approach by measuring fibrous materials’ dielectric properties as an auxiliary method.

Characterizing the dielectric properties can be classified into two types: non-resonant type^12–22^ and resonant type^22–27^. The former includes the transmission/reflection method and terahertz time-domain spectroscopy (THz TDS)^12–22^. The advantages of the non-resonant type are broadband with relatively low sensitivity. For the resonant type, the cavity perturbation method is the common choice for characterizing material’s complex permittivity in a specific frequency interval. Still, it has a defect that lossy materials will cause significant errors on measurement results^23,24^. Unfortunately, the lossless PAN fibers will turn into low-loss intermediates after the oxidation, and become lossy fibers after the pre-carbonization. Therefore, the enhanced-field method (EFM)^25,26^, together with a contour-mapping technique^27^, provides an accurate measurement of the complex permittivities of fibrous materials. The proposed method can serve as a diagnostic method to determine the processing status of the fibers by examining their dielectric properties.

## Results

**Figure 1:**
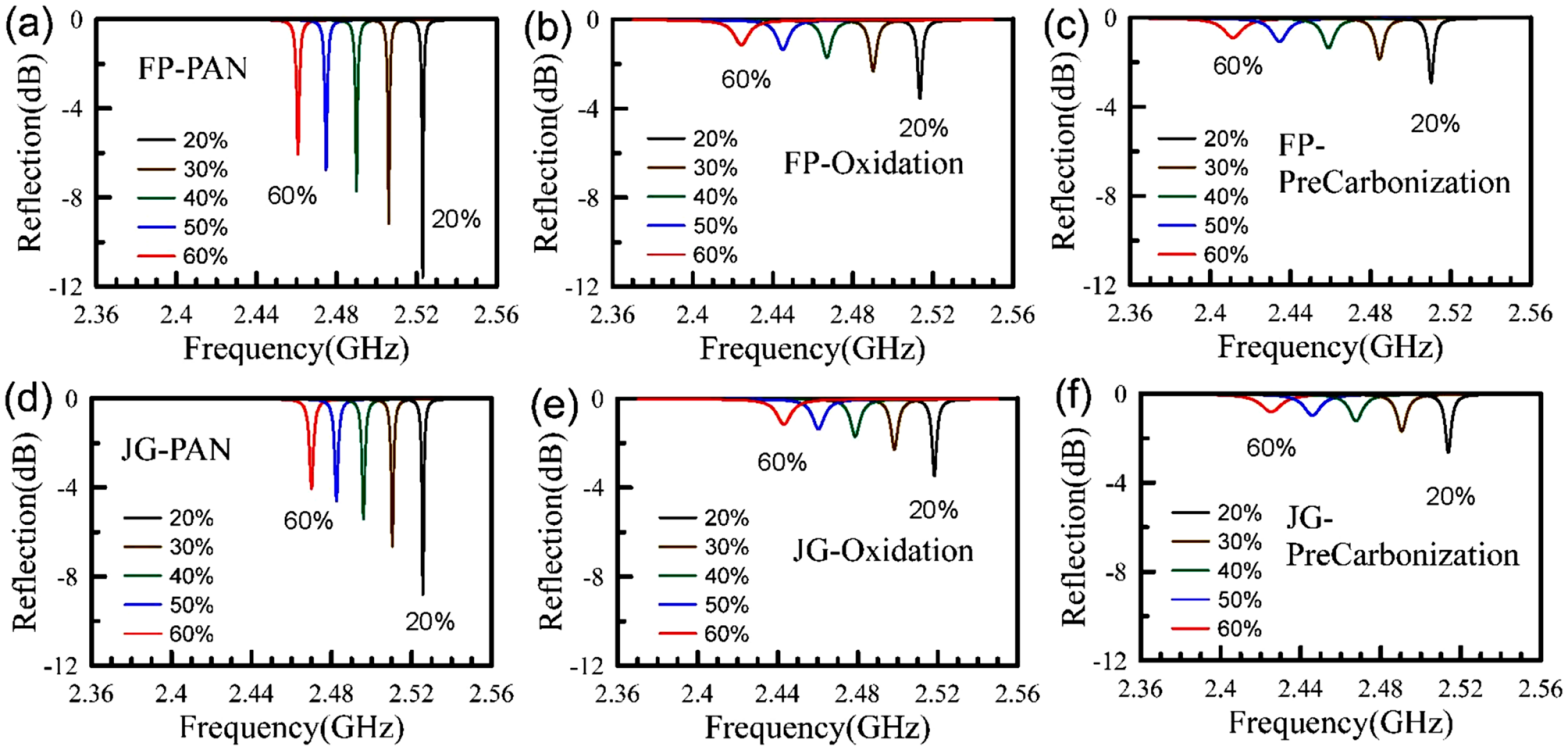
The measured reflection responses using EFM for Formosa Plastic in three stages: (**a**) PAN, (**b**) oxidation, and (**c**) pre-carbonization; and Jing Gong in three stages: (**d**) PAN, (**e**) oxidation, and (**f**) pre-carbonization. In each part, five different volumetric percentages from 20 to 60% are shown.

The dielectric properties of fibrous materials are challenging to measure because of the ultra-high aspect ratio (the length divided by the diameter)^28^. To overcome this problem, we cut the fibrous materials into pieces of 2–3 mm and cram them into a Teflon sample holder. The holder with fibrous materials inside is placed in a resonant cavity using EFM^25,26^. The EFM employs a single-port cavity. The reflection can be measured using a vector network analyzer (Anritsu, MS46122A). Figure 1 shows the measured reflections for three processing stages: PAN, oxidation, and pre-carbonization. For each part, five volumetric percentages from 20 to 60% are measured. Below the volumetric percentage of 20%, the fibers are not randomly scattered in Teflon holder, resulting in unwanted experimental errors. Above 60%, cramming the fibers into the holder becomes extremely difficult. That’s why we focus our attention on the volumetric percentages of 20–60%. The materials are obtained from two companies: Formosa Plastic Corporation (shown in Fig. 1a–c) and Jing Gong Co., Ltd. (shown in Fig. 1d–f).

As shown in Fig. 1, the resonant frequencies are inversely proportional to the volumetric concentrations. The FWHMs (full width at half maximum) of all reflected signals become border and border, which indicates that the quality factors (*Q*-factors) are also inversely proportional to the volumetric concentrations. Note that although the network analyzer (MS46112A) is low-cost, its frequency resolution is 1 Hz with the stability of ± 1 ppm. The network analyzer allows us to determine the resonant frequencies and the quality factors accurately. The major challenge is to cram the fibrous materials into the Teflon holder.

**Figure 2:**
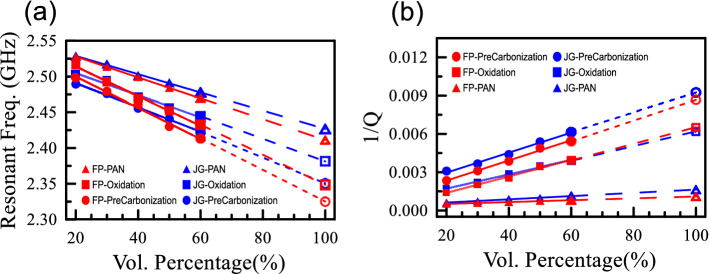
(**a**) Resonant frequencies and (**b**) reciprocals of *Q*-factors as functions of the volumetric percentages. The fibers at three stages from two companies are measured. The resonant frequencies are inversely proportional to the volumetric percentages, while the reciprocals of *Q*-factors are proportional to the volumetric percentages.

Figure 2 plots the resonant frequencies and the quality factors as functions of the volumetric concentrations for three processing stages: PAN, oxidation, and pre-carbonization, each with two suppliers. We can then extrapolate the resonant frequencies and the quality factors at 100% volumetric concentrations based on the observed linear trend. The effective medium method (extrapolated method) will be employed to determine the dielectric constants and loss tangents of carbon fibers with 100% volumetric percentage in different stages. The relations of resonant frequencies and volumetric percentages are plotted as Fig. 2a. The resonant frequencies are inversely proportional to volumetric percentages. But the relations between *Q*-factors and volumetric percentages are not simple. The relations of the reciprocals of *Q*-factors and volumetric percentages are plotted as Fig. 2b. The reciprocals of *Q*-factors are proportional to the volumetric percentages. Note that the reciprocal of a *Q*-factor is related to the loss tangents of the materials under test.

Table 1 shows the resonant frequencies and *Q*-factors of carbon fibers at the full volume for the PAN, oxidation, and pre-carbonization stages using the effective medium method. The vector network analyzer (MS46112A) can determine the resonant frequencies and the quality factors for each sample, accurately. The resonant frequency of PAN is higher than those of oxidation and pre-oxidation. Also, the *Q*-factor of PAN is higher than those of oxidation and pre-oxidation. Despite the quantitative difference in values, the samples from both companies exhibit the same trend. With the measured resonant frequencies and the *Q*-factors, we can then extract the complex permittivity.

**Table 1:** The extrapolated resonant frequencies and *Q*-factors of two brands with 100% volumetric percentages in the three stages: PAN, oxidation, and pre-carbonization.

	PAN		Oxidation		Pre-carbonization	
	Freq. (GHz)	*Q*-factor	Freq. (GHz)	*Q*-factor	Freq. (GHz)	*Q*-factor
Formosa Plastic	2.411 ± 0.001	910 ± 32	2.348 ± 0.009	153 ± 9	2.325 ± 0.014	115 ± 5
Jing Gong	2.427 ± 0.005	611 ± 35	2.381 ± 0.012	161 ± 7	2.351 ± 0.006	108 ± 5

The effective medium method suggests that the linear extrapolation should help us to find the resonant frequencies and *Q*-factors at 100% volumetric percentage. The fitted curve will be $y = mx + b$, where $y$ represents the resonant frequencies or the *Q*-factors, $x$ is the volumetric percentage. $m$ and $b$ are the slope and the $y$-intercept. Due to the measuring uncertainty, there will be the mean squared error (MSE). The mean squared error will give rise to the errors of the regression parameters, i.e., $\Delta m$ and $\Delta b$, which will then contribute to the worst errors of $\Delta y$ when $x = 1$. According to the propagation of error for a linear model, the extrapolated errors, $\Delta y = \pm (\sqrt {\Delta m^{2} + \Delta b^{2} } )$, are listed in Table 1. The deviations of the resonant frequencies are relatively small as expected, but the errors of the *Q*-factors are non-negligible which will influence the accuracy of the imaginary permittivity as discussed below.

The real and imaginary parts of the complex permittivity ($\varepsilon = \varepsilon^{\prime} + i\varepsilon^{\prime\prime}$) are coupled through the Kramers–Kronig relations. The resonant frequency and the quality factor will be functions of the real part and the imaginary part of the permittivity, i.e.,

1 $$f_{r} = f_{r} (\varepsilon^{\prime}, \, \varepsilon^{\prime\prime}),$$

2 $$Q = Q(\varepsilon^{\prime}, \, \varepsilon^{\prime\prime}).$$

For lossless materials, $\varepsilon^{\prime}$ and $\varepsilon^{\prime\prime}$ can be determined independently^25,26^. The real part of the complex permittivity is related to the resonant frequency, it reads $\varepsilon^{\prime} = \varepsilon^{\prime}(f_{r} )$. But for the imaginary permittivity $\varepsilon^{\prime\prime}$, it still depends on both $f_{r}$ and $Q$. Since $f_{r}$ helps us determine the real permittivity, we can express $\varepsilon^{\prime\prime} = \varepsilon^{\prime\prime}(Q, \, \varepsilon^{\prime})$. However, for lossy materials, $\varepsilon^{\prime}$ and $\varepsilon^{\prime\prime}$ are closely coupled, the contour mapping technique is introduced to find the correct values^27^. The real permittivity is commonly normalized and expressed as the relative dielectric constant ($\varepsilon_{r} = {{\varepsilon^{\prime}} \mathord{\left/ {\vphantom {{\varepsilon^{\prime}} {\varepsilon_{0} }}} \right. \kern-\nulldelimiterspace} {\varepsilon_{0} }}$). For the expression of the imaginary permittivity, it is normally denoted as the loss tangent ($\tan \delta = {{\varepsilon^{\prime\prime}} \mathord{\left/ {\vphantom {{\varepsilon^{\prime\prime}} {\varepsilon^{\prime}}}} \right. \kern-\nulldelimiterspace} {\varepsilon^{\prime}}}$). Equations (1) and (2) can be rewritten as:

3 $$f_{r} = f_{r} (\varepsilon_{r} ,{\text{ tan}}\delta ),$$

4 $$Q = Q(\varepsilon_{r} ,{\text{ tan}}\delta ).$$

**Figure 3:**
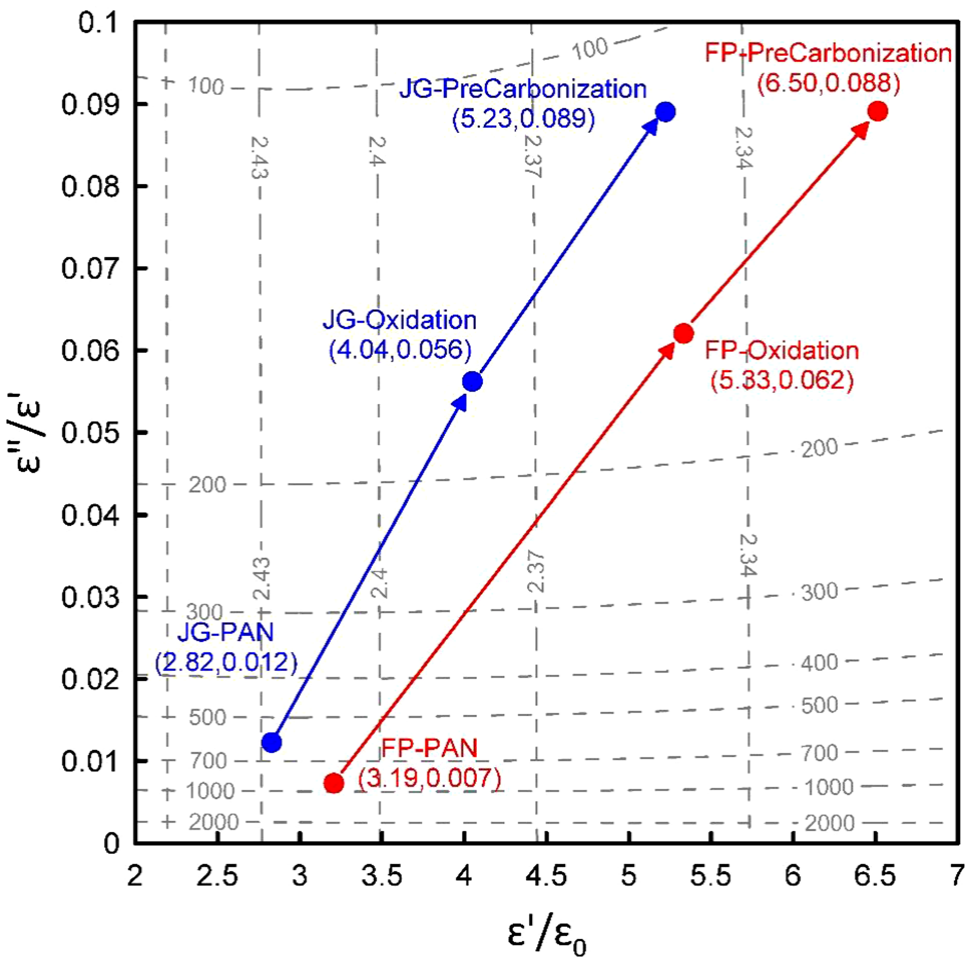
The contour map determines the relative dielectric constant and the loss tangent. Two categories of isolines are obtained from HFSS simulation. Iso-resonant frequency lines and iso-*Q*-factor lines are plotted with dashed lines. From the measured data as shown in Table 1, we can uniquely determine the relative dielectric constant and the loss tangent as marked in the figure and listed in Table 2.

The HFSS (High-Frequency Structure Simulator, ANSYS Inc.) provides reliable simulation results at microwave frequency. By changing the input parameters $\varepsilon_{r}$ and $\tan \delta$ of the proposed cavity. A contour map is plotted in Fig. 3. Figure 3 verifies our understanding that for low-loss materials ($\tan \delta < 0.01$), the resonant frequency depends merely on the real permittivity, $f_{r} = f_{r} (\varepsilon^{\prime})$ or $\varepsilon^{\prime} = \varepsilon^{\prime}(f_{r} )$.

The measured resonant frequency $f_{r}$ and quality factor $Q$ in Table 1 can be used to find the corresponding $\varepsilon_{r} = {{\varepsilon^{\prime}} \mathord{\left/ {\vphantom {{\varepsilon^{\prime}} {\varepsilon_{0} }}} \right. \kern-\nulldelimiterspace} {\varepsilon_{0} }}$ and $\tan \delta = {{\varepsilon^{\prime\prime}} \mathord{\left/ {\vphantom {{\varepsilon^{\prime\prime}} {\varepsilon^{\prime}}}} \right. \kern-\nulldelimiterspace} {\varepsilon^{\prime}}}$. For example, the resonant frequency and *Q*-factor of the PAN stage for Formosa Plastic are 2.411 GHz and 910. By mapping the contour lines of $f_{r}$ and $Q$, we obtain an intersection point at $\varepsilon_{r} = 3.19$ and $\tan \delta = 0.007$. The corresponding material’s properties ($\varepsilon_{r}$, $\tan \delta$) obtained from the measured data set ($f_{r}$, $Q$) are marked in Fig. 3 and listed in Table 2.

**Table 2:** The measured dielectric constants and loss tangent for the two brands at 100% volumetric percentage for three stages: PAN, oxidation, and pre-carbonization.

	PAN		Oxidation		Pre-carbonization	
	Dielectric const.	Loss tangent	Dielectric const.	Loss tangent	Dielectric const.	Loss tangent
Formosa Plastic	3.19 ± 0.001	0.007 ± 0.0002	5.33 ± 0.019	0.062 ± 0.0035	6.50 ± 0.038	0.089 ± 0.0042
Jing Gong	2.82 ± 0.006	0.012 ± 0.0007	4.04 ± 0.021	0.056 ± 0.0024	5.23 ± 0.014	0.089 ± 0.0044

Table 2 shows that the relative dielectric constant ($\varepsilon_{r}$) and the corresponding loss tangent ($\tan \delta$) increase as the processing temperature rises. For both companies (Formosa Plastic and Jing Gong), the PANs have the lowest dielectric constant and loss tangent, while the pre-carbonization fibers have the highest values. The loss tangent is generally related to the color of the material. A white or light yellow material is not a good microwave absorber with low $\tan \delta$, while a brown or black one is usually a good microwave absorber with high $\tan \delta$. Besides, the fibers shrink in size as the processing temperature rises, indicating that the densities of fibers increase. The increasing dielectric constant seems to be related to the increasing density. However, the increasing density is unable to explain the changes of the loss tangent. As the processing temperature increases, the fibers go through severe chemical reactions, not just physical densification.

The complex permittivities of the fibers from the two companies have distinct values, but, interestingly, they have a similar trend. The better the carbonization, the higher the $\varepsilon_{r}$ and $\tan \delta$. In other words, characterizing the $\varepsilon_{r}$ and $\tan \delta$ helps to evaluate the carbonization status of a PAN fiber. Note that the proposed approach is an auxiliary method, at least for now. It should be used together with other diagnostic techniques, such as FTIR, DSC, Raman spectrum, SEM, and XRD.

## Discussions

### Conductivity effect

The PAN is a dielectric material with extremely low electric conductivity, but the final product, i.e., the carbon fiber, is a good conductor under direct current (DC). This indicates that the conductivity of a carbonized fiber changes from extremely low (insulator) to high (conductor) during the processing stages^10^. It is essential to know the applicability of the proposed dielectric characterizing method.

The generalized dielectric constant contributes from the bound charges and the free charges, as shown below.

5 $$\varepsilon (\omega ) = 1 + \frac{{e^{2} }}{m}\sum\limits_{{j({\text{bound}})}} {\frac{{Nf_{j} }}{{(\omega_{j}^{2} - \omega^{2} - i\omega \gamma_{j} )}}} + i\frac{{e^{2} Nf_{0} }}{{m\omega (\gamma_{0} - i\omega )}},$$

where $Nf_{j}$ is the number of bound electrons of the *j*-th kind per unit volume and $Nf_{0}$ is the number of free electrons per unit volume. $\omega$ is the driving frequency of the wave. $\omega_{j}$ and $\gamma_{j}$ are the natural resonant frequency and the collision frequency of the *j*-th kind bound electrons, respectively. For free electrons, $\omega_{0} = 0$ and $\gamma_{0}$ is much greater than $\omega$. For an ideal insulator like PAN, the contribution from the free electrons is zero because $Nf_{0} = 0$. The second term of Eq. (5) can be omitted. Therefore, the complex dielectric constant of an insulator is $\varepsilon = \varepsilon^{\prime}_{b} + i\varepsilon^{\prime\prime}_{b}$, which considers the contribution of the bound charges only.

When the number density of the free electrons is non-zero, i.e., $Nf_{0} \ne 0$, Eq. (5) can be rewritten as:

6 $$\varepsilon (\omega ) = \varepsilon^{\prime}_{b} + i\varepsilon^{\prime\prime}_{b} + i\frac{\sigma }{\omega },$$

where $\sigma = {{e^{2} Nf_{0} } \mathord{\left/ {\vphantom {{e^{2} Nf_{0} } {m\gamma_{0} }}} \right. \kern-\nulldelimiterspace} {m\gamma_{0} }}$ with the condition $\gamma_{0} \gg \omega$. Equation (6) tells that,

7 $$\varepsilon_{r} = \frac{{\varepsilon^{\prime}_{b} }}{{\varepsilon_{0} }},$$

8 $$\tan \delta = \frac{{\varepsilon^{\prime\prime}_{b} + \frac{\sigma }{\omega }}}{{\varepsilon^{\prime}_{b} }}.$$

The conductivity of the carbonized fiber ranges from 0.1 to 1000 Siemens*/*m^1,2,10^. The resonant frequency is around 2.45 GHz and the angular frequency $\omega = 1.54 \times 10^{10} {\text{ rad/s}}$. Even though at the case of the optimal conductivity, i.e., $\sigma = 1000{\text{ S/m}}$, we still have

9 $$\varepsilon^{\prime\prime}_{b} \gg \frac{\sigma }{\omega }.$$

The conductivities at the early stages of the fabrication process, i.e., PAN, oxidation, and pre-carbonization, are low. It is fair and reasonable to claim that the contribution from the conductivity is negligible, which warrants the applicability of the proposed method. However, the authors admit that whether the proposed method is applicable to the carbonization/graphitization fibers is still under investigation, which is beyond the scope of this work.

It is worth noting that comparing with the dielectric constant measured in Ref.^29^, our measured dielectric constant is much smaller than theirs. The reason is that the fibers under study are anisotropic, and they measured the axial dielectric properties of the carbon fiber. Our measured fibers are much shorter and randomly orientated. In other words, our measurement provides an averaged dielectric properties of fibrous material and can be used to characterize the carbonization status.

## Method

### Enhanced-field method (EFM)

**Figure 4:**
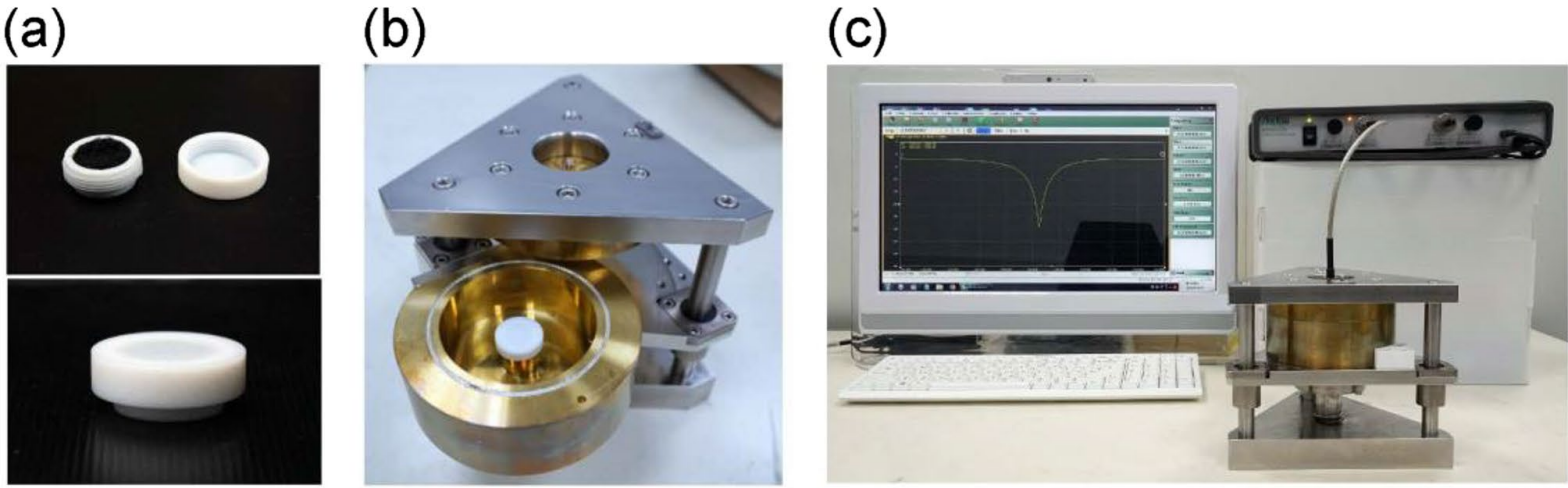
Experimental setup of the enhanced-field method (EFM). (**a**) The fiber is crammed randomly into a Teflon sample holder. (**b**) The sample holder is then placed in the specially designed EFM cavity at a frequency around 2.45 GHz. (**c**) The one-port system is connected to a network analyzer to measure the frequency response of the reflectivity.

The enhanced-electric field provides a stronger coupling with the dielectric properties of the specimens, resulting in a better resolution in characterization than that of the conventional perturbation method^25–27^. The specimens to be measured are PAN, oxidized, and pre-carbonized fibers. All of them are provided by two companies: Formosa Plastic Corporation and Jing Gong Co., Ltd. The fibers are cut into small pieces of 2–3 mm, randomly packed, and crammed into a Teflon holder, as shown in Fig. 4a. The outer diameter of the Teflon holder is *ϕ* 22.00 mm with a height of 9.00 mm. The inner diameter of the holder is *ϕ* 14.00 mm with a height of 7.00 mm. The capacity of the holder is 1.078 cm^3^. The Teflon holder is placed at the center of the cavity (Fig. 4b) and measured the frequency response with a vector network analyzer (VNA) in a range from 2.35 GHz to 2.6 GHz. The resonant frequency and quality factor can be derived from the measured frequency response (Fig. 4c).

The samples to be measured are fibrous with an extremely high aspect ratio. The traditional permittivity measurements require a sample with a fixed/specific shape. Here we solve the problem by cramming the fibers into the Teflon sample holder. The diameter of the fiber is around 5–10 μm. The free space wavelength of 2.45 GHz is 12 cm. Since the wavelength is several orders higher than the diameter of the fibers, the nonuniformity in the sheath and core regions is averaged. In other words, the proposed technique is unable to resolve the sheath-core problem. Besides, the length of the fibers is around 2 mm, and the fibers are randomly packed in three dimensions. This warrants an averaged effect. The proposed technique is advantageous to samples with amorphous or non-fixed shapes, like liquids, flakes, and powders.

### Density of the fibers

**Figure 5:**
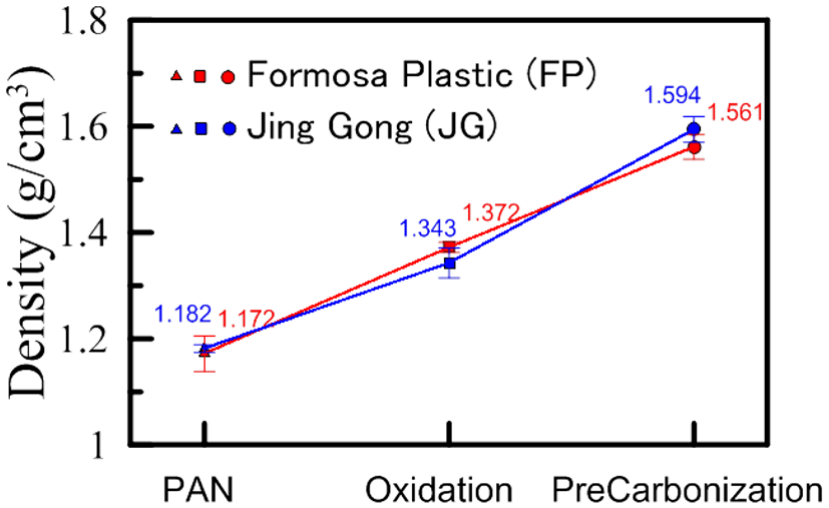
The densities of the fibers for three stages: PAN, oxidation, and pre-carbonization. The materials under test are obtained from Formosa Plastic Corporation (FP, red symbols) and Jing Gong Co., Ltd. (JG, blue symbols).

Evaluating the volumes of powders or fibrous materials is challenging, as compared with the measurement of the volumes of solid or liquid samples. The volume ($V$) of a fibrous sample is associated with the mass ($m$) and the density ($\rho$), i.e., $V = {m \mathord{\left/ {\vphantom {m \rho }} \right. \kern-\nulldelimiterspace} \rho }$. The fibers are lightweight materials. They must be weighted with a precision balance. The density of fibrous materials is difficult to obtain in that the radii and lengths of the fibers vary greatly. Archimedes’ principle is employed to evaluate the densities of the fibers. During the density measurement, the air bubbles should be completely removed. The measured densities are displayed in Fig. 5. The radii and lengths of the fibers shrink as the processing temperature increases. Because of the densification effect, the densities of the fibers will increase with the processing temperature. Once again, the samples obtained from two companies are displayed side-by-side. Their trend is quite similar, with minor differences in values. The measured values agree well with our expectations and can be used to calculate the samples’ volumetric concentrations.
